# Novel cuproptosis-related genes *C1QBP* and *PFKP* identified as prognostic and therapeutic targets in lung adenocarcinoma

**DOI:** 10.1515/biol-2025-1142

**Published:** 2025-09-30

**Authors:** Yanju Lv, Xiaozhuo Duan, Xueli Yuan

**Affiliations:** Department of Oncology, The Fourth Affiliated Hospital of Harbin Medical University, Harbin, 150000, China; Department of Oncology, The Second Affiliated Hospital of Harbin Medical University, Harbin, 150000, China

**Keywords:** cuproptosis, LUAD, *C1QBP*, *PFKP*

## Abstract

Excessive intracellular copper accumulation triggers cuproptosis, a novel regulated cell death process with therapeutic potential. Analyzing 566 The Cancer Genome Atlas samples alongside lung adenocarcinoma (LUAD)-specific microarray and single-cell sequencing data, we identified 109 cuproptosis-associated genes, of which *C1QBP* and *PFKP* emerged as key prognostic markers. Four-gene risk model stratified patients into high- and low-risk groups with distinct survival outcomes, where high-risk scores correlated with advanced TNM stages. Clinical validation confirmed that elevated *C1QBP*/*PFKP* expression in LUAD tissues predicted shorter progression-free survival. Functional assays demonstrated that silencing *C1QBP* or *PFKP* increased intracellular copper concentration, suppressed proliferation, and inhibited invasion, mechanistically linking these genes to cuproptosis dysregulation. Our findings nominate *C1QBP*/*PFKP* as actionable targets for LUAD therapy, offering both prognostic biomarkers and copper-metabolism-directed treatment strategies.

## 1 Introduction

Lung cancer is the most prevalent cancer globally and a major cause of cancer-related deaths around the world. Non-small-cell lung cancer makes up more than 80% of all lung cancer cases, with lung adenocarcinoma (LUAD) being the most frequently occurring subtype [1]. The northern region is characterized by a cold climate and heavy industry, and studies have shown that air pollution and cold weather are associated with a higher incidence of lung cancer [2,3]. This combination of environmental and industrial factors creates a unique risk profile for lung cancer in the northern region. Despite significant improvements in treatment strategies for LUAD in recent years, the prognosis remains poor [4].

Cuproptosis is a novel regulatory mechanism of apoptotic cell death that occurs due to the excessive accumulation of copper ions in cells, leading to mitochondrial lipid peroxidation and alterations in iron-sulfur clusters [5,6,7]. Unlike apoptosis (programmed cell death) and ferroptosis (iron-dependent lipid peroxidation), cuproptosis is driven by irreversible mitochondrial copper accumulation, leading to distinct aggregation of lipoylated tricarboxylic acid (TCA) cycle proteins and catastrophic energy collapse PMID: 38801962.

Numerous studies have reported that intracellular copper can participate in the development of various tumors through multiple mechanisms, suggesting it could be a potential therapeutic target [6]. However, the mechanisms regulating cuproptosis in tumor cells in LUAD remain unclear, necessitating urgent research into therapeutic targets targeting this regulatory mechanism. Beyond cancer, cuproptosis plays established roles in neurodegenerative diseases – notably Alzheimer’s, where copper chelation therapy is under clinical evaluation (PMID: 35691251). In cardiovascular systems, copper-dependent cardiomyocyte death exacerbates heart failure progression. These findings underscore the broad pathophysiological relevance of copper homeostasis (PMID: 36774340).

This study identified 109 prognostically significant cuproptosis-related genes using single-factor Cox regression analysis. After excluding conflicting genes, we selected 11 key genes for further analysis. Finally, we validated the tissue expression and *in vitro* functions of two genes, *C1QBP* and *PFKP*, which were found to be involved in copper ion metabolism in LUAC and affect tumor cell proliferation and invasion capabilities.

## 2 Materials and methods

### 2.1 Data collection

We acquired RNA-seq data in TPM format along with clinical information for LUAD from The Cancer Genome Atlas (TCGA) database, selecting 566 samples, which include 59 normal and 507 LUAD tissues. In addition to the TCGA data, we included LUAD-related microarray and single-cell sequencing data specifically collected from northern region patients, which were downloaded from the NCBI GEO database (GSE13213, GSE31210, GSE37745, GSE68465, and GSE72094). Clinical details for these datasets are summarized in Table S1.

### 2.2 Screening of cuproptosis-related genes

We studied 347 cuproptosis-related genes with false discovery rate < 0.05, as detailed in Table S2. Expression data for these genes were extracted from the TCGA dataset and integrated with follow-up information. Univariate Cox regression analysis, performed with the R package “survival,” identified prognostic genes related to cuproptosis. The risk and favorable genes were compared using the R package “VennDiagram” [8].

### 2.3 Development and validation of the risk prediction model

Univariate Cox regression analysis identified prognostic genes in the training group. Additionally, multivariate Cox regression was conducted, with variable selection guided by the Akaike information criterion (AIC), using the R package “survival. Risk scores were calculated using the “predict” function from the R “stats” package, and patients were divided into high-risk and low-risk groups according to the median risk score. Prognostic differences were assessed using the survfit function and the log-rank test. The R package “timeROC” was used to calculate the area under the receiver operating characteristic (ROC) curve (AUC) for 1, 2, and 3 years. The model was validated using internal test groups, external datasets (GSE13213, GSE31210, GSE68465, GSE72094), and the TCGA dataset to ensure accuracy and robustness.

### 2.4 Immune infiltration and immunotherapy analysis

The levels of immune cell infiltration were assessed using the CIBERSORT algorithm [9]. The R package “estimate” assessed stromal, immune, and ESTIMATE scores to evaluate the tumor microenvironment. Immune phenotype scores (IPS) predicted patient responses to immunotherapy and provided insights into tumor immune characteristics [10].

### 2.5 Clinical sample collection

Clinical samples were collected from LUAD patients who had been confirmed by histopathology and were scheduled for surgery at the hospital. None of these patients had undergone prior systemic anti-tumor therapy. Tumor and adjacent normal tissues, each approximately 1 cm³, were excised during surgery, immediately fixed in formalin for immunohistochemistry, and stored in liquid nitrogen for quantitative PCR (qPCR).


**Informed consent:** Informed consent has been obtained from all individuals included in this study.
**Ethical approval:** The research related to human use has been complied with all the relevant national regulations and institutional policies and in accordance with the tenets of the Helsinki Declaration and has been approved by the authors’ institutional review board or equivalent committee.

### 2.6 Immunohistochemistry

Immunohistochemical analysis of clinical LUAD and adjacent normal tissues was performed to detect *C1QBP*, *PFKP*, and cuproptosis markers (*FDX1*, *LIAS*, *HSP70*) using the DAB staining method. The expression levels of these proteins were quantified and analyzed for their association with cuproptosis markers.

### 2.7 qPCR

qPCR was conducted on LUAD tissues, adjacent normal tissues, and A549 cell lines before and after siRNA interference. This analysis quantified mRNA levels of *C1QBP*, *PFKP*, and cuproptosis markers (*FDX1*, *LIAS*, *HSP70*) to validate their role in cuproptosis regulation in LUAD. The primer sequences (5′−3′) were as follows:

*FDX1*-F CTGGCTTGTTCAACCTGTCACC

*FDX1*-R GATTTGGCAGCCCAACCGTGAT

*LIAS*-F GCCAAGAAGGTTCAGCCTGATG

*LIAS*-R GTCTACATCTGCCTCACGAAGTG

*HSP70*-F GACCTGCCAATCGAATCAGC

*HSP70*-R CTGCGTTCTTAGCATCATTCCGC

*PFKP*-F CCAGTCCAGAGATGTGCCG

*PFKP*-R TGGCCGAAGATGAAGAGCG

*C1QBP*-F GCTGCTTATGGAGATGGACAA

*C1QBP*-R CCAGGACACAGAGGCAACA

Gene expression was calculated using the 2^−ΔΔCt^ method with GAPDH as endogenous control. Reactions were performed in triplicate under the following conditions: 95°C for 30 s, followed by 40 cycles of 95°C for 5 s and 60°C for 30 s using QuantStudio 6 Pro system (Applied Biosystems).

### 2.8 Cell culture and treatment

The A549 LUAD cell line was purchased from the National Center for Model Organisms/Chinese Academy of Sciences Cell Bank. Cells were cultured in DMEM supplemented with 10% fetal bovine serum and 1% penicillin–streptomycin under 5% CO_2_ at 37°C. Gene silencing of *C1QBP* and *PFKP* was achieved using RNA interference (RNAi). siRNA sequences (5′−3′) were as follows:

*PFKP*: GCAGAACUCUUCAAUGAUATT; UAUCAUUG AAGAGUUCUGCTT

*C1QBP*: GGAAGAUGCCUCUGAUUAUTT; AUAAU AGAGGCAUCUUCCTT

Cells were transfected with 50 nM siRNA using Lipofectamine 3000 (Invitrogen) for 48 h. Scramble siRNA (5′-UUCUCCGAACGUGUCACGU-3′) served as a negative control. Knockdown efficiency was verified at 24, 48, and 72 h post-transfection by Western blotting.

### 2.9 Intracellular copper ion concentration detection

Intracellular copper ion concentrations in A549 cells, both before and after *C1QBP* and *PFKP* interference, were measured using a complexometric and colorimetric method. The copper assay kit (Elabscience) was used for the experiment. The copper ion concentration (μmol/g protein) was calculated using the following formula (1):

(1) \[\hspace{-2em}\text{Copper}\hspace{.3em}\text{ion}\hspace{.3em}\text{concentration}=(\mumol/\text{gprot})\hspace{3em}=(\Delta A580-b)/a\times f/\text{Cpr,}\hspace{-1em}]\]

where Δ*A*580 is the OD value of the standard well − OD value of the blank well (OD value when the standard concentration is 0), *a* = slope of the standard curve, *b* is the intercept of the standard curve, *f* is the dilution factor of the sample before adding to the detection system, and Cpr is the protein concentration of the sample before adding to the detection system (g protein/L)

The standard curve is fitted by the following formula:

(2) \[y=ax+b,]\]

where *y* is the OD value of the standard well − OD value of the blank well (OD value when the standard concentration is zero) and *x* is the concentration of the standard.

### 2.10 Cell function assays

Cell viability was evaluated using the cell counting kit-8 (CCK8) assay to investigate the effects of *C1QBP* and *PFKP* on A549 cells. Proliferation was measured with the BeyoClick™ EdU-488 Cell Proliferation Detection Kit. Clonogenic ability was evaluated through a plate cloning assay, and cell migration was assessed using the Transwell invasion assay.

### 2.11 Image analysis

Images were acquired using an Olympus BX53 microscope (400× magnification). Scale bars were calibrated with ImageJ (v1.53) and inserted using Adobe Illustrator. Arrows annotate regions discussed in the text.

## 3 Results

### 3.1 Identification of cuproptosis-related prognostic genes in LUAD

We performed a univariate Cox regression analysis to identify genes linked to prognosis in LUAD patients, resulting in 109 cuproptosis-related genes. Among these, 106 were identified as risk factors and 3 as favorable factors (Table S3). After excluding conflicting genes *REXO2* and *COQ7*, we categorized the remaining 345 genes into two groups: negative (*n* = 31) and positive (*n* = 314) target genes.

**Figure 1:**
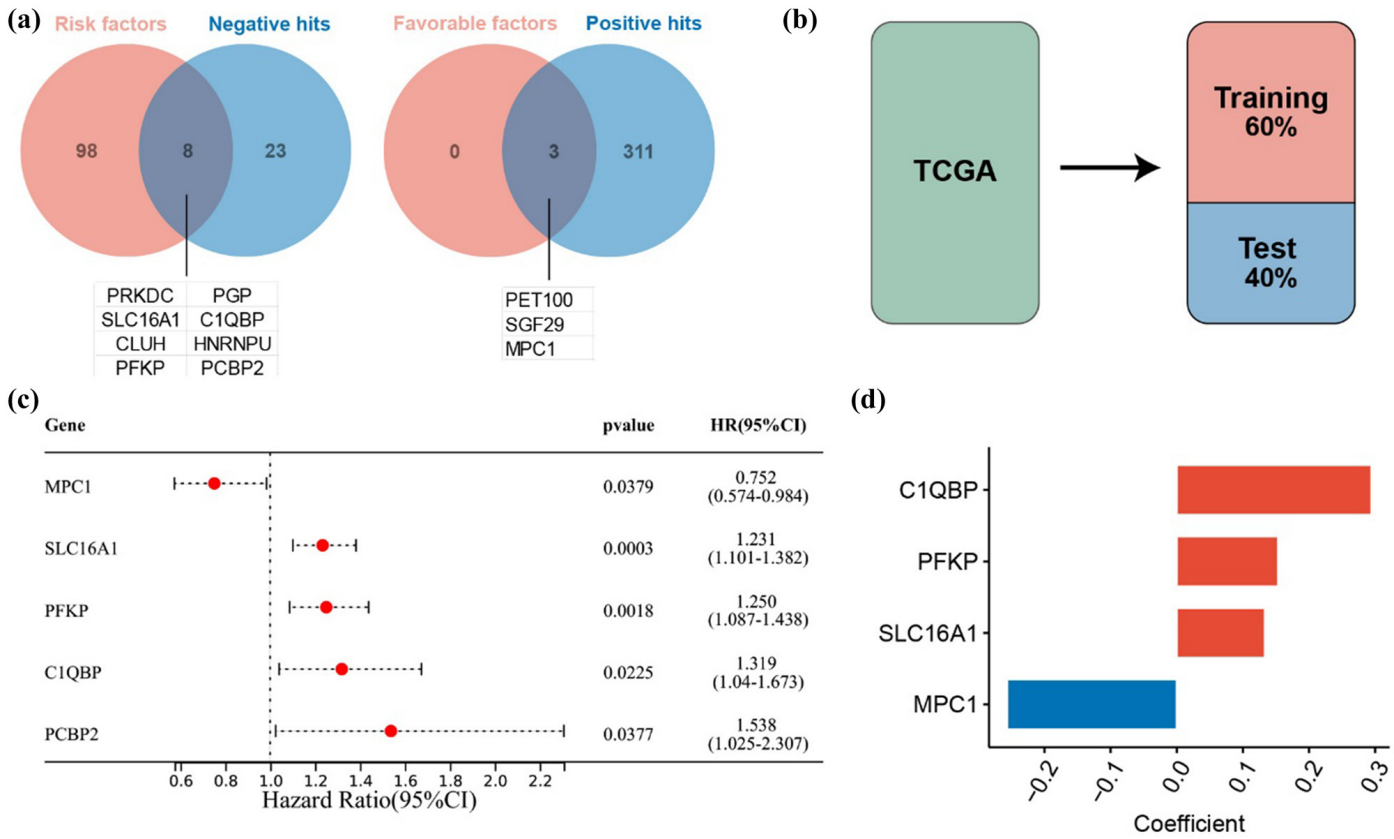
Establishment of the prognostic model for cuproptosis-related genes. (a) Venn diagram showing the intersection of prognostic genes and cuproptosis-related genes. (b) Division of the TCGA cohort into training and testing groups. (c) Univariate Cox regression analysis based on the training group. (d) Regression coefficients of the model genes.

We then intersected the risk factors with the negative target genes, identifying eight genes: *PRKDC*, *PGP*, *SLC16A1*, *C1QBP*, *CLUH*, *HNRNPU*, *PFKP*, and *PCBP2*. Intersecting favorable factors with positive target genes yielded three genes: *PET100, SGF29*, and *MPC1* (Figure 1a). Thus, we identified 11 cuproptosis-related genes for further exploration of their roles in LUAD progression.

### 3.2 Development of a prognostic model using cuproptosis-related genes

**Figure 2:**
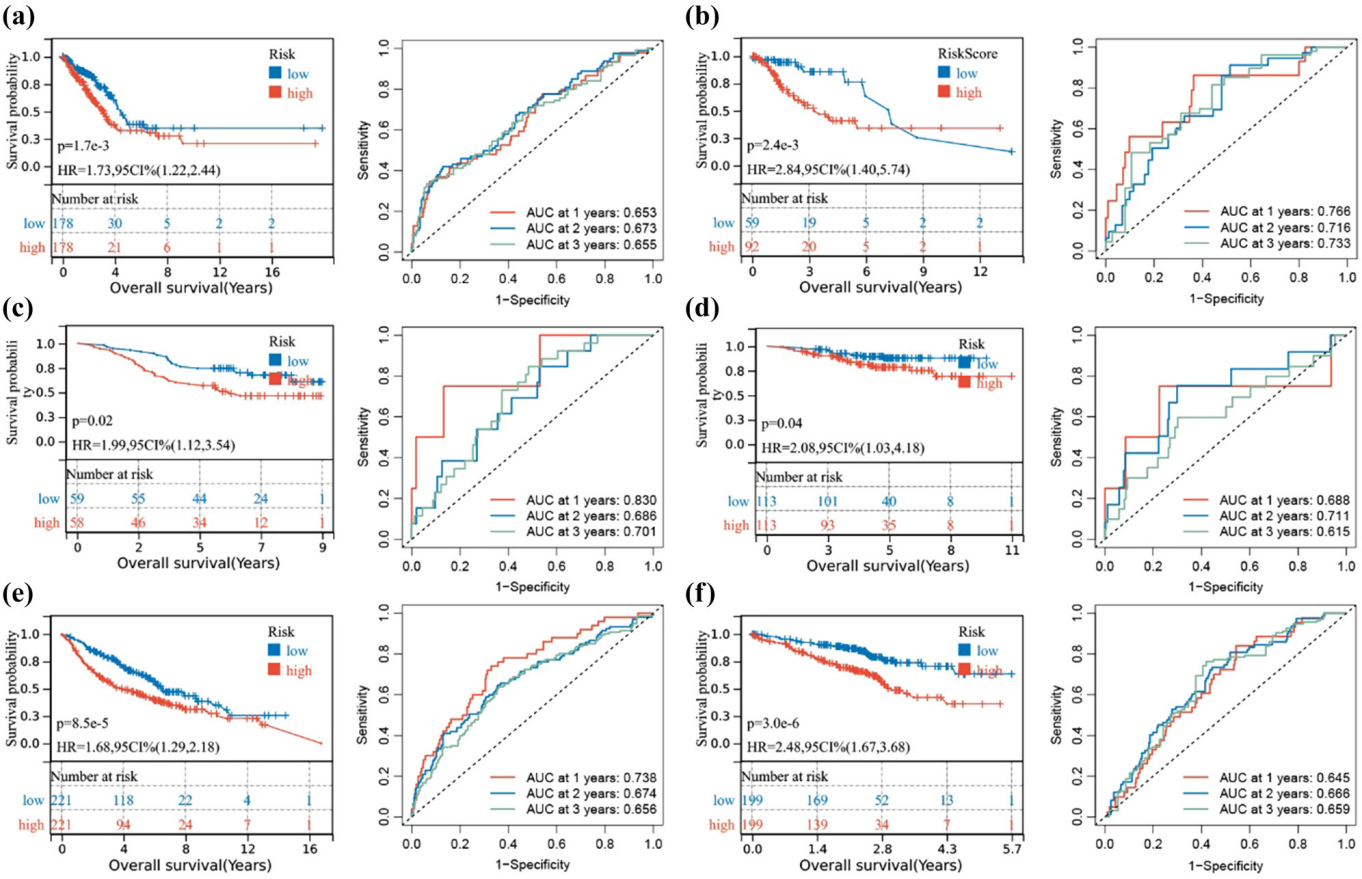
Prognostic analysis of the model. The predictive ability of the model was validated using Kaplan–Meier survival analysis and ROC curves in (a) the training group, (b) the internal testing group, (c) the GSE13213 dataset, (d) the GSE31210 dataset, (e) the GSE68465 dataset, and (f) the GSE72094 dataset.

The TCGA dataset was randomly divided into two groups: 60% for training and 40% for testing, in order to develop a prognostic model (Figure 1b). Univariate Cox regression in the training group identified five candidate genes associated with overall survival (OS) (Figure 1c). Multivariate Cox regression with AIC selection finalized the model genes as *C1QBP*, *PFKP*, *SLC16A1*, and *MPC1* (Figure 1d). Patients were divided into high-risk and low-risk groups according to the median risk score. The model demonstrated significant prognostic differentiation between these groups (Figure 2a), achieving AUC values of 0.653, 0.673, and 0.655 for 1-, 2-, and 3-year assessments, respectively.

### 3.3 Internal testing and external validation of the prognostic model

Validation within the internal testing group revealed that high-risk patients experienced significantly lower OS (*P* = 0.0024). The 1-, 2-, and 3-year AUC values were 0.766, 0.716, and 0.733, respectively (Figure 2b). The model’s robustness was confirmed across external datasets, including GSE13213, GSE31210, GSE68465, and GSE72094, where high-risk patients also exhibited poorer survival rates (Figure 2c–f). These validations affirm the model’s stability and predictive accuracy across diverse datasets.

### 3.4 Analysis of risk scores and clinical characteristics

**Figure 3:**
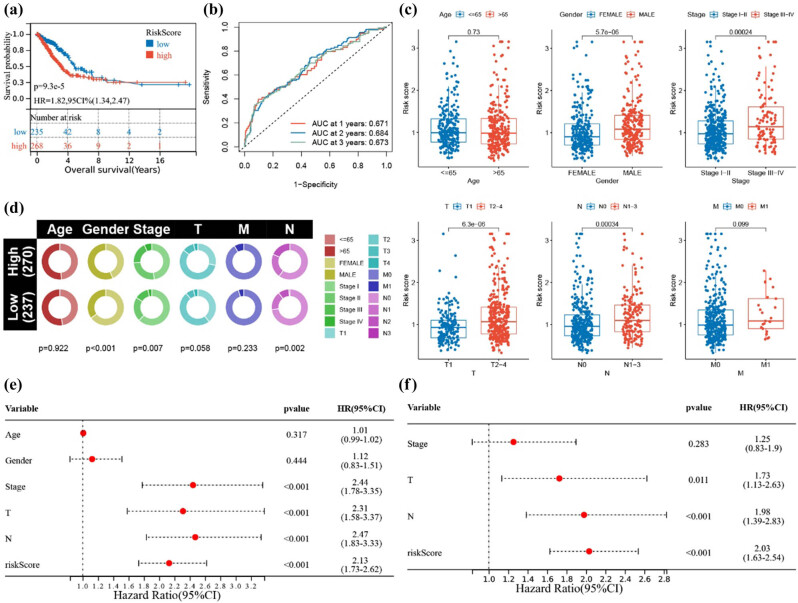
Analysis of risk score and clinical characteristics. (a) Kaplan–Meier curves of patients in high and low-risk groups in the TCGA dataset. (b) ROC curves of the model in the TCGA dataset. (c) Differential analysis of RiskScore across clinical characteristic groups (including age, gender, stage, T stage, N stage, M stage). (d) Distribution of clinical characteristic groups, including age, gender, stage, T stage, M stage, and N stage. (e) Univariate Cox regression analysis. (f) Multivariate Cox regression analysis.

In both training and testing groups of the TCGA dataset, high-risk scores correlated with significantly lower OS (*P* < 0.001), with 1-, 2-, and 3-year AUC values of 0.671, 0.684, and 0.673 (Figure 3a and b). High-risk scores were associated with male gender (*P* < 0.001), advanced disease stages (*P* < 0.001), and lymph node involvement (*P* < 0.001) (Figure 3c). High-risk patients were significantly more prevalent among males (*P* < 0.001), those with advanced disease (*P* = 0.007), and those with lymph node involvement (*P* = 0.002) (Figure 3d). Risk scores were confirmed as a significant prognostic factor (hazard ratio [HR] = 2.13, *P* < 0.001) and as an independent prognostic indicator (HR = 2.13, *P* < 0.001) (Figure 3e and f).

### 3.5 Immune microenvironment and immunotherapy

**Figure 4:**
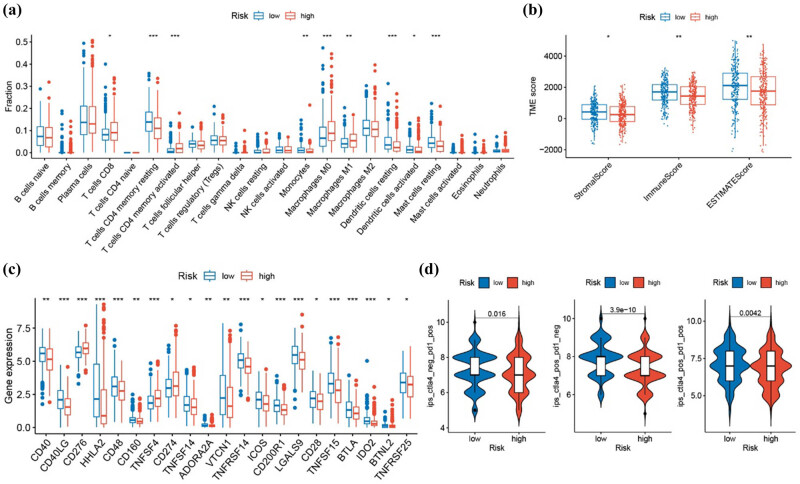
Immune infiltration and immunotherapy analysis. (a) Analysis of 22 immune cells using CIBERSORT. (b) Analysis of immune infiltration using ESTIMATE. (c) Differential expression of immune checkpoint genes between high and low-risk groups. (d) IPS prediction of immunotherapy efficacy. *P ≤ 0.05, **P ≤ 0.01, ***P ≤ 0.001.

The CIBERSORT algorithm showed increased infiltration of CD8+ T cells, activated memory CD4+ T cells, and M0 and M1 macrophages in high-risk patients. In contrast, there was a decrease in resting memory CD4+ T cells, monocytes, resting mast cells, and dendritic cells (Figure 4a). The ESTIMATE algorithm indicated that the high-risk group had lower stromal, immune, and ESTIMATE scores (Figure 4b). The expression of immune checkpoint genes was generally higher in the low-risk group, except for CD276, TNFSF4, and CD274 (Figure 4c). IPS suggested that patients in the low-risk category might benefit more from immunotherapy (Figure 4d).

### 3.6 Upregulation of *C1QBP* and *PFKP* in LUAD

**Figure 5:**
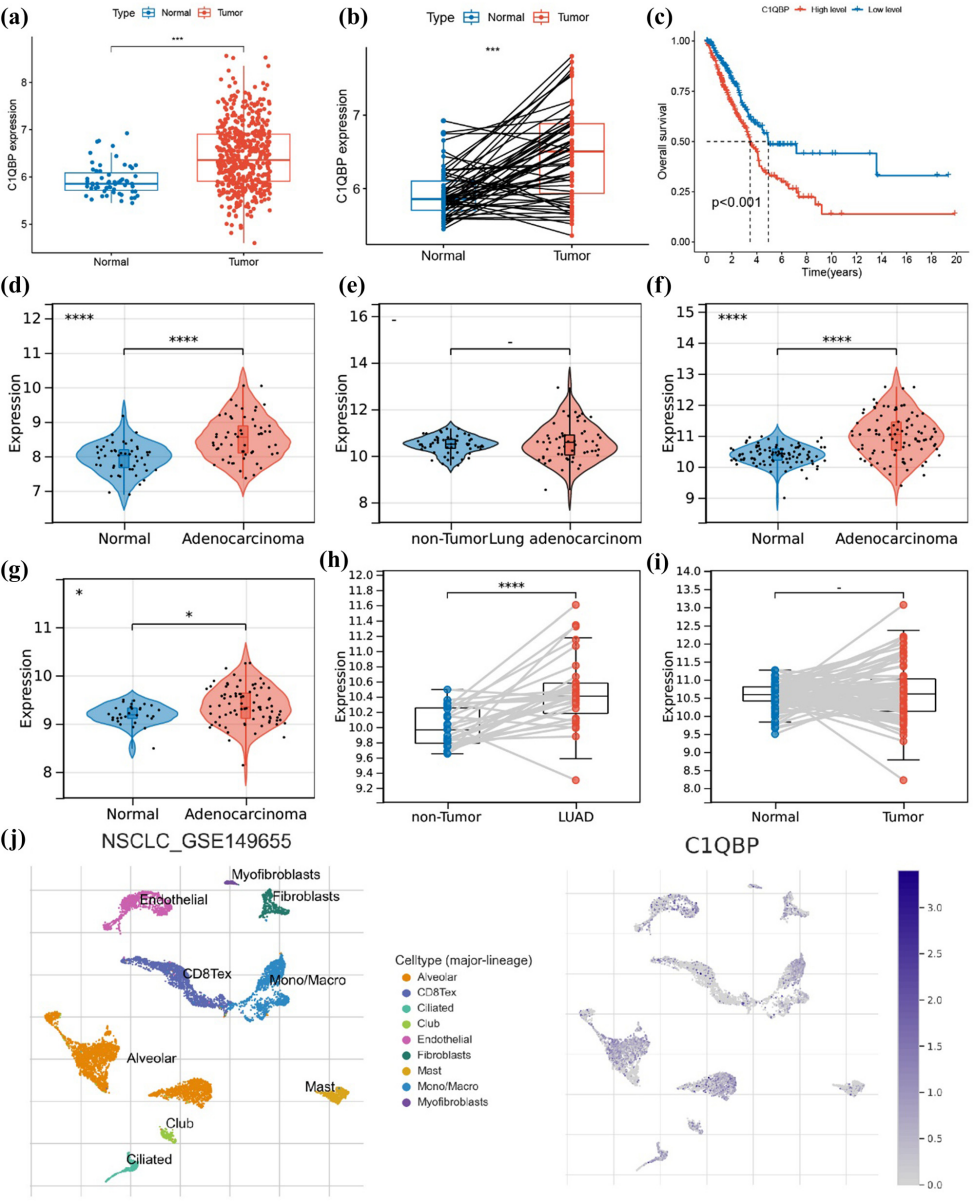
Upregulation of *C1QBP* in LUAD tissues. (a) Differential expression of *C1QBP* in the TCGA dataset. (b) Differential expression of *C1QBP* in paired samples from the TCGA dataset. (c) KM curve of *C1QBP* in the TCGA dataset. (d) Expression of *C1QBP* in the GSE10072 dataset, (e) GSE32863 dataset, (f) GSE40791 dataset, (g) GSE43458 dataset, (h) GSE63459 dataset, and (i) GSE75037 dataset. (j) Analysis of the GSE149655 dataset using the TISCH database. *P ≤ 0.05, ***P ≤ 0.001, ****P ≤ 0.0001.

The expression of *C1QBP* was significantly upregulated in LUAD tissues compared to adjacent non-cancerous tissues (*P* < 0.001) and associated with worse prognosis (*P* = 0.018) (Figure 5a–c). This trend was consistent across six GEO datasets (Figure 5d–i). Analysis of the GSE149655 single-cell sequencing dataset in the TISCH online database (http://tisch.comp-genomics.org/home/) revealed *C1QBP* expression predominantly in alveolar cells, monocytes/macrophages, and endothelial cells (Figure 5j).

**Figure 6:**
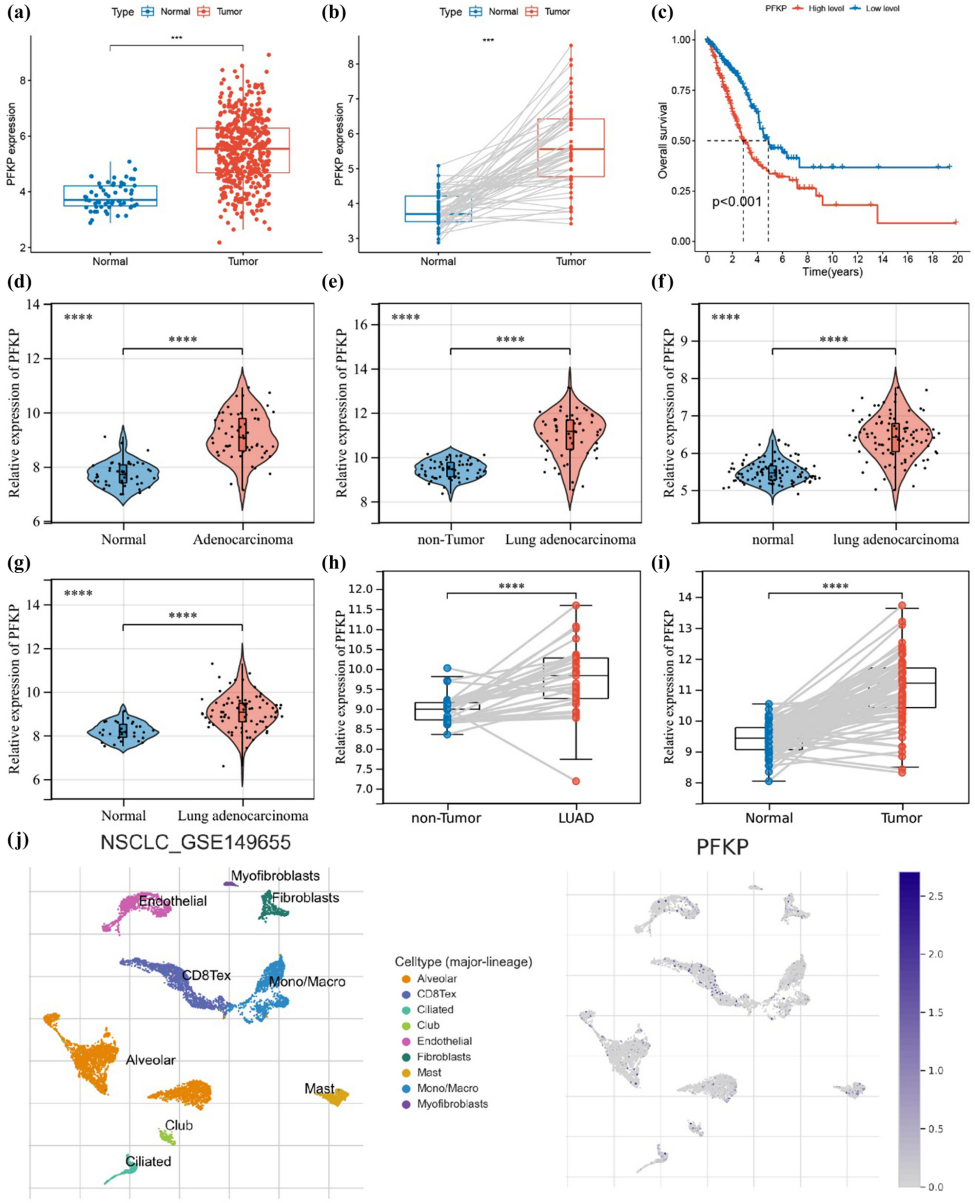
Upregulation of *PFKP* in LUAD tissues. (a) Differential expression of *PFKP* in the TCGA dataset. (b) Differential expression of *PFKP* in paired samples from the TCGA dataset. (c) KM curve of *PFKP* in the TCGA dataset. (d) Expression of *PFKP* in the GSE10072 dataset, (e) GSE32863 dataset, (f) GSE40791 dataset, (g) GSE43458 dataset, (h) GSE63459 dataset, and (i) GSE75037 dataset. (j) Analysis of the GSE149655 dataset using the TISCH database. ***P ≤ 0.001, ****P ≤ 0.0001.

*PFKP* was also significantly upregulated in LUAD tissues compared to adjacent tissues (*P* < 0.001) and associated with poor prognosis (*P* = 0.018) (Figure 6a–c). This upregulation was confirmed across six GEO datasets (Figure 6d–i). Single-cell analysis showed *PFKP* primarily in CD8+ T cells and endothelial cells (Figure 6j).

### 3.7 Differential expression of cuproptosis-related genes *C1QBP* and *PFKP* in LUAD tissues

**Figure 7:**
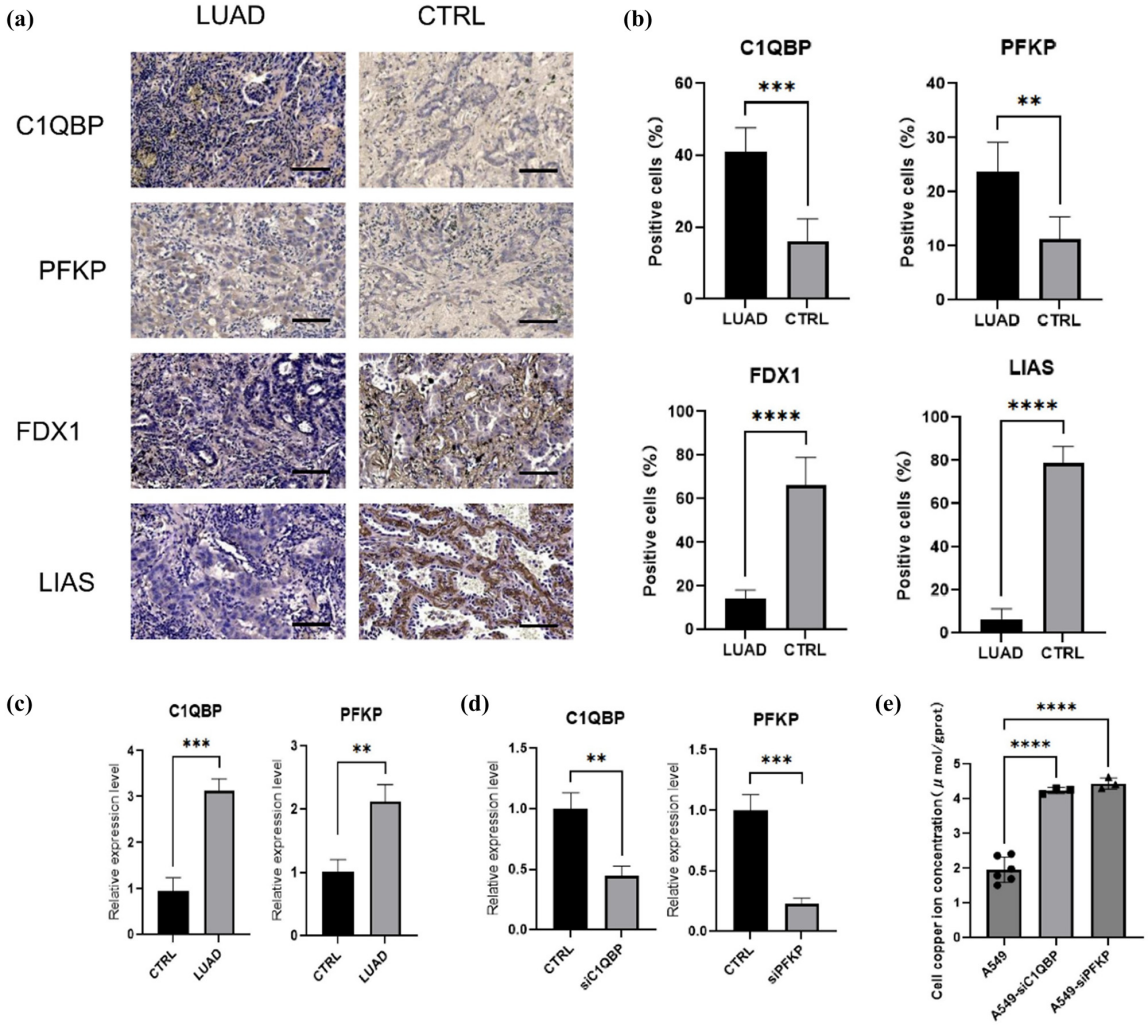
The cuproptosis related genes *C1QBP* and *PFKP* are differentially expressed in LUAD tissues. (a) Representative IHC images of C1QBP in LUAD (scale bar: 100μm, arrows indicate positively stained tumor cells). (b) Quantification of staining intensity (mean ± SEM, *n* = 5, *P < 0.05 by Student’s *t*-test). (c) Relative mRNA expression levels of target genes *C1QBP* and *PFKP* and cuproptosis marker genes *FDX1* and *LIAS*. Adenocarcinoma of the lung tumor tissue (LUAD), control paracancerous tissue (CTRL). *N* = 3, *t*-test, *P ≤ 0.05. (d) Relative mRNA expression before and after RNA interference. *N* = 3, *t*-test, *P ≤ 0.05. (e) Cell copper ion concentration. One-way analysis of variance test, A549 *N* = 5, A549-siC1QBP *N* = 3, A549-siPFKP *N* = 3, *P ≤ 0.05. **P ≤ 0.01, ***P ≤ 0.001, ****P ≤ 0.0001.

Molecular biology experiments on tumor tissues from 5 LUAD patients revealed higher expression of *C1QBP* and *PFKP* in tumors compared to adjacent tissues (Figure 7a and b). *PFKP* was highly expressed in the extracellular matrix of tumors, while *C1QBP* was more cytoplasmic in tumors. Cuproptosis markers *FDX1* and *LIAS* were less expressed in tumors but prominent in adjacent tissues. qPCR validated the higher mRNA expression levels of *C1QBP* and *PFKP* in tumor tissues (Figure 7c).

### 3.8 Regulation of copper ion concentration by *C1QBP* and *PFKP*

Previous research has shown that copper-induced cell death results from the accumulation of copper ions, which disrupts iron-sulfur cluster proteins and induces stress responses related to protein toxicity [6]. Key measurable indicators include intracellular copper ion concentration, pyruvate, α-ketoglutarate, *FDX1*, *DLAT*, *LIAS*, and *HSP70* [5]. In this study, we used FDX1 and *LIAS* as detection targets for copper-induced cell death to investigate the role of *C1QBP* and *PFKP* in LUAD *in vitro*.

RNAi in A549 cells confirmed successful silencing of *C1QBP* and *PFKP* (Figure 7d). Intracellular copper ion concentrations were significantly higher in A549-si*C1QBP* and A549-si*PFKP* cells compared to original A549 cells, indicating that downregulation of these proteins increases copper ion levels (Figure 7e). This suggests that *C1QBP* and *PFKP* might influence LUAD progression by modulating cuproptosis signaling. Using the copper colorimetric assay kit, we detected the copper ion concentrations as shown in Tables S4 and S5.

### 3.9 Regulation of cuproptosis markers by *C1QBP* and *PFKP*

**Figure 8:**
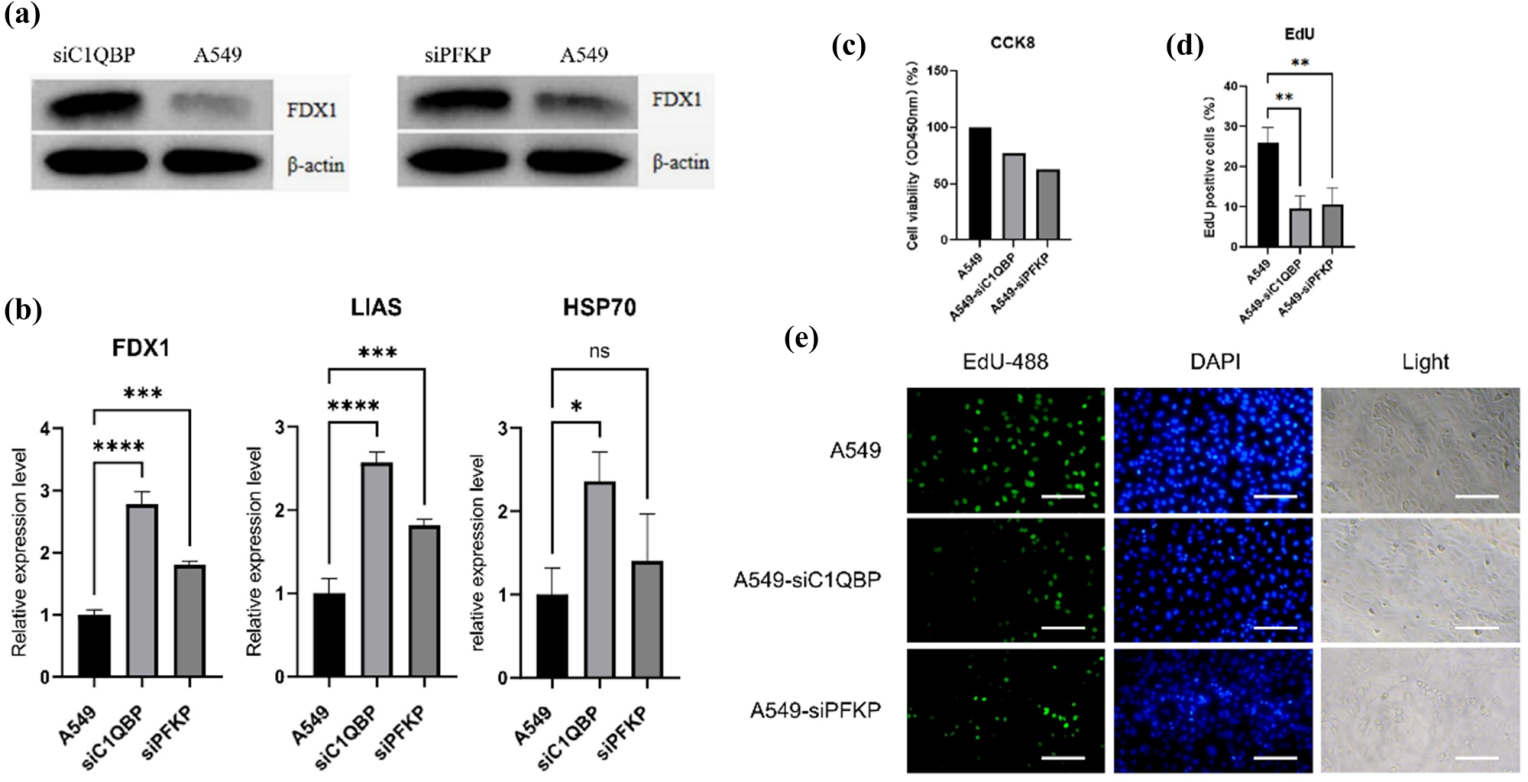
*C1QBP* and *PFKP* can regulate cuproptosis. (a) Western blot of *FDX1* in A549, A549-siC1QBP and A549-siPFKP. (b) Relative expression of cuproptosis maker genes mRNA in A549, A549-siC1QBP and A549-siPFKP. One-way analysis of variance (ANOVA) test, A549 *N* = 3, A549-siC1QBP *N* = 3, A549-siPFKP *N* = 3, *P ≤ 0.05. (c) Cell viability of A549, A549-siC1QBP and A549-siPFKP. (d) EdU-positive cell percentage. One-way ANOVA test, *N* = 3, *P ≤ 0.05. (e) EdU fluorescence staining of A549, A549-siC1QBP and A549-siPFKP. **P ≤ 0.01, ***P ≤ 0.001, ****P ≤ 0.0001.

To further investigate the potential functions of *C1QBP* and *PFKP* in LUAD, we conducted additional experiments using the A549 LUAD cell line. Western blot analysis showed elevated *FDX1* protein levels in A549-si*C1QBP* and A549-si*PFKP* cells compared to original A549 cells (Figure 8a and b), indicating increased cuproptosis levels following downregulation of *C1QBP* and *PFKP*.

### 3.10 Effect of *C1QBP* and *PFKP* on cell proliferation and invasion

**Figure 9:**
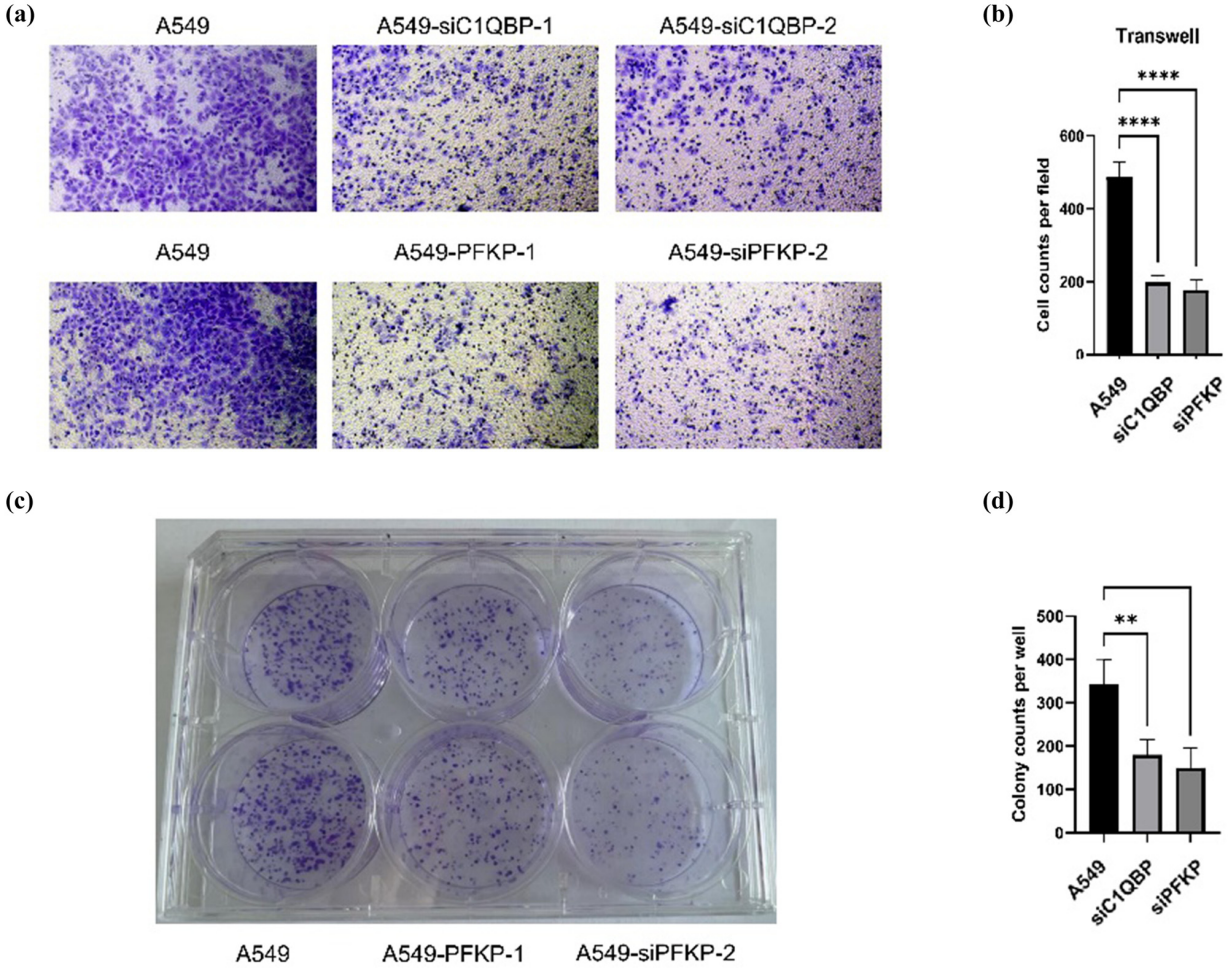
*C1QBP* and *PFKP* can regulate the *in vitro* proliferation and invasion of LUAD cell. (a) Images of cells in the inner chamber of the Transwell. (b) Cell counts per field by Transwell. One-way analysis of variance (ANOVA) test, *N* = 3, ***P ≤ 0.0001. (c) Images of cell clone formation. (d) Cell clone formation. One-way ANOVA test, *N* = 3, **P ≤ 0.01.

CCK8 assays revealed significantly lower cell viability in A549-si*C1QBP* and A549-si*PFKP* cells (Table S6, Figure 8c). EdU assays showed reduced DNA replication activity in these cells (Figure 8d and e). Transwell assays demonstrated decreased invasion ability in *C1QBP* and *PFKP* interference groups (Figure 9a and b), and colony formation assays showed fewer colonies (Figure 9c and d). These results suggest that *C1QBP* and *PFKP* regulate LUAD cell proliferation and invasion.

## 4 Discussion

Tumor progression is characterized by the uncontrolled growth of malignant cells, which often exhibit heightened metabolic demands compared to normal cells. This increased demand for nutrients and oxygen leads to severe deficiencies in the tumor microenvironment, which in turn impacts tumor survival and growth [11]. Copper, a crucial cofactor for numerous enzymes, plays a vital role in maintaining cellular functions [7,12]. Under normal conditions, copper concentrations are tightly regulated to avoid toxic accumulation, thereby preserving cellular balance [13].

Recent studies have introduced a novel concept in copper metabolism: copper-dependent cell death [14]. This form of cell death differs from traditional programmed cell deaths like ferroptosis and apoptosis. Copper directly interacts with lipid components in the TCA cycle, causing acylated proteins to aggregate and resulting in the loss of iron-sulfur cluster proteins. This process leads to protein toxicity and ultimately cell death [14]. This discovery highlights the importance of copper homeostasis and its impact on immune cell infiltration.

The notion of “copper-dependent cell proliferation” has emerged, describing how copper regulates tumor growth through both enzymatic and non-enzymatic mechanisms [15]. However, when copper levels exceed a critical threshold, it becomes toxic, inducing a type of cell death referred to as “cuproptosis.” This process involves copper-induced disruptions in mitochondrial pathways, suggesting a potential therapeutic strategy for cancer [6].

Our study utilized RNA-seq data and clinical information from TCGA and the NCBI GEO database, focusing on 347 cuproptosis-related genes. We identified 109 genes with prognostic significance through univariate Cox regression analysis, from which 11 key genes were selected after excluding contradictory ones.

We developed a prognostic model using TCGA data, dividing the dataset into training and testing groups. Univariate and multivariate Cox regression analyses identified four significant genes (*C1QBP*, *PFKP*, *SLC16A1*, and *MPC1*). The model effectively categorized patients into high-risk and low-risk groups based on median risk scores, with high-risk patients showing significantly poorer OS in both the training and validation groups. The model’s robustness was confirmed across external datasets (GSE13213, GSE31210, GSE68465, GSE72094).

The risk score correlated with clinical features, revealing higher scores in male patients, those with advanced stages, and those with lymph node metastasis. Cox regression analysis revealed that the risk score is an independent predictor of prognosis. Immune infiltration analysis showed increased CD8+ T-cell and M0/M1 macrophage infiltration in high-risk patients, while low-risk patients exhibited higher stromal and immune scores [PMID: 38773982] [PMID: 39664584]. Immune phenotype scoring suggested that patients classified as low-risk may have a better response to immunotherapy. Our findings revealed that *C1QBP* and *PFKP* were upregulated in LUAD tissues and linked to a poorer prognosis. Single-cell analysis confirmed their differential expression across various cell types within LUAD tissues. *PFKP*’s role in tumors involves regulating energy metabolism and influencing glycolytic pathways, which are crucial under hypoxic conditions. High *PFKP* expression enhances glycolysis, supporting rapid tumor cell proliferation and invasion [16,17]. *C1QBP*, on the other hand, regulates immune responses, oxidative stress, and cell cycle control and is associated with intracellular redox balance and oxidative stress responses [18,19,20,21].

Experimental validation of *C1QBP* and *PFKP* revealed their higher expression in LUAD tissues than in adjacent non-cancerous tissues. Silencing these genes increased the expression of cuproptosis markers (*FDX1*, *LIAS*), suggesting their involvement in cuproptosis regulation. Additionally, reduced cell proliferation and invasion were observed in cells with silenced *C1QBP* and *PFKP*, indicating their potential role as oncogenes.

Combining bioinformatics, histological, and *in vitro* data, we hypothesize that *C1QBP* and *PFKP* regulate LUAD progression through cellular cuproptosis mechanisms. These findings provide important insights into potential therapeutic strategies that focus on copper metabolism for cancer treatment. Further research is needed to explore how manipulating intracellular copper levels can affect tumor biology and provide new avenues for LUAD therapy. Therapeutic inhibition of *C1QBP/PFKP* may be achievable using existing strategies: *PFKP* is targeted by metabolic inhibitors like 3PO in preclinical models [PMID: 38473963], while *C1QBP* interacts with chemosensitizers such as obatoclax [citation]. Copper chelators could synergize with these approaches to induce cuproptosis, though combinatorial efficacy requires testing [PMID: 28107702] [PMID: 39664584].

This study has the following limitations. First, while the model shows predictive value (AUC 0.65–0.73), we acknowledge this modest performance may limit standalone clinical application. However, our risk score could complement existing biomarkers through multi-modal integration, similar to how inflammatory scores enhance prognostic systems like TNM staging. Future studies should validate combinatorial approaches. Second, while our data demonstrate copper-dependent *C1QBP/PFKP* associations – supported by *FDX1/LIAS* correlations – we recognize these are indirect measures of cuproptosis. Direct validation of copper-induced mitochondrial dysfunction remains an important goal for future studies. Third, while immunohistochemistry validated the overexpression of *C1QBP/PFKP* in LUAD tissues (*n* = 5), the small sample size restricts generalizability. Larger-scale pathological studies are needed to confirm the spatial distribution of these proteins across tumor stages and subtypes.

In summary, while research on cuproptosis-related genes in LUAD is still evolving, *C1QBP* and *PFKP* appear to play significant roles in tumor progression. Understanding the concentration-specific effects of copper on normal versus tumor cells could reveal new therapeutic strategies for LUAD. The insights gained from this study pave the way for future research into targeted cuproptosis therapies in cancer treatment.

## 5 Conclusion

*C1QBP* and *PFKP* are critical cuproptosis-related genes in LUAD, with their high expression linked to poor prognosis and increased tumor progression; targeting these genes could offer potential therapeutic strategies for managing LUAD.

## Supplementary Material

Supplementary Table 1

Supplementary Table 2

Supplementary Table 3

Supplementary Table 4

Supplementary Table 5

Supplementary Table 6
